# MiR‐149 sensitizes esophageal cancer cell lines to cisplatin by targeting DNA polymerase β

**DOI:** 10.1111/jcmm.13659

**Published:** 2018-05-04

**Authors:** Yuanyuan Wang, Jiahui Chen, Man Zhang, Wenwen Zhang, Min Li, Wenqiao Zang, Ziming Dong, Guoqiang Zhao

**Affiliations:** ^1^ College of Basic Medical Sciences Zhengzhou University Zhengzhou Henan China; ^2^ Collaborative Innovation Center of Cancer Chemoprevention of Henan Zhengzhou Henan China

**Keywords:** chemotherapy, DNA polymerase β, esophageal cancer, miR‐149

## Abstract

Human DNA polymerase β (polβ) is a small, monomeric protein essential for short‐patch base excision repair (BER). polβ plays an important role in the regulation of chemotherapy sensitivity in tumour cells. In this study, we determined that the expression levels of polβ mRNA and miR‐149 in tumour tissues were significantly higher than in adjacent non‐tumour tissues. We also found that the expression level of miR‐149 in EC tumour tissues was inverse to that of polβ expression. Bioinformatics analysis and dual‐luciferase reporter assay predicted that miR‐149 negatively regulates polβ expression by directly binding to its 3′UTR. CCK‐8 assay indicated that miR‐149 could enhance the anti‐proliferative effects of cisplatin in EC1 and EC9706 cell lines. Flow cytometry, caspase 3/7 activity, and immunofluorescence microscopy results indicated that miR‐149 could enhance the apoptotic effects of cisplatin in EC1 and EC9706 cell lines. We also showed that the expression of polβ lacking the 3′UTR sequence could override the proliferative and apoptotic functions of miR‐149, suggesting that miR‐149 negatively regulates polβ expression by binding to its 3′UTR. Surface plasmon resonance results also showed that miR‐149 could bind with wild‐type polβ. In addition, we identified a new variant of polβ (C1134G). In conclusion, this study confirms that miR‐149 may enhance the sensitivity of EC cell lines to cisplatin by targeting polβ, and that miR‐149 may be unable to regulate the C1134G variant of polβ. Based on these findings, potential drugs could be developed with a focus on enhanced sensitivity of EC patients to chemotherapy.

## 1 INTRODUCTION

MicroRNAs (miRNAs) are a class of small, noncoding regulatory RNAs that are approximately 18‐24 nucleotides in length. MicroRNAs negatively regulate gene expression at the post‐transcriptional and translational level by triggering cleavage of target mRNAs, or inhibiting protein translation via sequence‐specific interaction with the 3′‐untranslated regions (3′‐UTRs) of target mRNAs.^1^, ^2^, ^3^, ^4^, ^5^, ^6^ miRNAs are reported to be intrinsic regulators of many cellular processes such as cell invasion, differentiation, proliferation and apoptosis.^7^, ^8^, ^9^, ^10^, ^11^, ^12^ Therefore, aberrant expression of miRNAs may lead to the development and progression of cancer, and have prognostic significance for several tumour types.^13^, ^14^, ^15^, ^16^

DNA polymerase β (polβ) is a member of the DNA polymerase family and is essential for base excision repair (BER), one of the major pathways of DNA repair.^17^, ^18^, ^19^, ^20^, ^21^ Thirty percent of all tumours reported to date harbour mutations in the polβ gene.^22^ Aberrant polβ expression results in an increased rate of spontaneous mutagenesis, and a highly mutagenic phenotype.^23^, ^24^ Studies have reported polβ mutations in various cancer types, and have shown that this may play a role in mediating tumour sensitivity to cisplatin.^25^, ^26^, ^27^, ^28^, ^29^

Esophageal cancer (EC) is a major cause of cancer‐related deaths worldwide. Many previous studies have reported that the polβ gene is often mutated in primary EC tissues. EC also exhibits varying degrees of sensitivity to chemotherapy in the clinic. Previously, we performed miRNA chip‐based expression analysis of EC tissues and found that the expression of miR‐149 in EC tissues was aberrant. Based on bioinformatic analyses, we hypothesized that the human polβ 3′UTR contains the putative binding sites for miR‐149, and that miR‐149 may affect the sensitivity of EC cell lines to cisplatin.

In this study, we first investigated whether miR‐149 modulates polβ expression, and then examined the influence of miR‐149 on cisplatin sensitivity in EC cell lines. We identified a novel homozygous C to G point mutation at nucleotide 1134 (C1134G) in the polβ gene of EC patient tissues, and analysed the relationship between C1134G polβ and miR‐149.

## 2 MATERIALS AND METHODS

### 2.1 Patients and tissue specimens

Specimens were collected from a total of 82 EC patients with TNM stage III between 2011 and 2015, from the First Affiliated Hospital of Zhengzhou University and the Oncology Hospital of Linzhou City. All specimens were obtained using endoscopy and biopsy assays. Patients received chemotherapy with cisplatin (100 mg/m^2^ body surface area; Day 1) and 5‐FU (1000 mg/m^2^ body surface area; Days 1‐5), repeated every 28 days; none had received chemotherapy or radiotherapy prior to surgery. The patients were followed for a minimum of 36 months. All patients were informed in advance and signed explicit informed consent forms. This study was approved by the ethics committee of Zhengzhou University.

### 2.2 RNA extraction and quantitative real‐time PCR

Total RNA was isolated from biopsy EC tissues and adjacent non‐tumour tissue samples using TRIzol reagent (Invitrogen, Carlsbad, CA, USA) according to the manufacturer's instructions. miR‐149 expression level was obtained using quantitative real‐time PCR (qRT‐PCR) assay with high‐specificity miR‐149 qRT‐PCR Detection Kit (Stratagene Corp, La Jolla, CA). U6 snRNA was used as normalization control for miR‐149. To determine polβ expression level, β‐actin was used as normalization control. HET‐1A cell line was used as untreated control to make the different groups comparable. The qRT‐PCR results were expressed as threshold cycle (Ct) and were converted to the fold change (2^−ΔΔCt^).

### 2.3 Cell lines

EC1, EC9706, and HET‐1A cells were purchased from the Type Culture Collection of the Chinese Academy of Sciences (Shanghai, China). All cells were maintained in RPMI 1640 medium supplemented with 10% foetal bovine serum (FBS; Gibco BRL, Gaithersburg, MD, USA) and incubated at 37°C and 5% CO_2_.

### 2.4 Western blotting

Total protein from cultured cells was extracted using RIPA buffer containing phenylmethanesulfonyl fluoride. Protein concentrations were determined with BCA protein assay kit (Beyotime, Haimen, China). Extracted proteins were subjected to SDS‐PAGE gels and then transferred to polyvinylidene difluoride membranes. After blocking, the membranes were incubated overnight at 4°C with diluted (1:1000) primary antibody (polyclonal rabbit anti‐polβ). Following extensive washing, the membranes were incubated with diluted (1:3000) horseradish‐peroxidase‐conjugated goat anti‐rabbit IgG (Santa Cruz Biotechnology). Signals were detected using a chemiluminescence detection kit (Amersham Pharmacia Biotech, Piscataway, NJ, USA). An antibody against β‐actin (Santa Cruz Biotechnology) served as an endogenous reference.

### 2.5 Plasmid construction and the luciferase reporter assay

The human polβ 3′untranslated region (UTR) fragment containing putative binding sites for miR‐149 was amplified from human genomic DNA via PCR. The mutant polβ 3′ UTRs were obtained by overlap extension PCR. The fragments were cloned into a pmirGLO reporter vector (Promega, Madison, WI, USA), downstream of the luciferase gene, to generate the recombinant vectors pmirGLO‐Wt and pmirGLO‐Mt.

For the luciferase reporter assay, EC1 and EC9706 cells were co‐transfected with miRNA (miR‐149 mimic or scrambled‐miR‐149 negative control; GenePharma, Shanghai, China) and reporter vectors (pmirGLO‐Wt reporter vectors or pmirGLO‐Mt reporter vectors), using a BTX ECM 2001 electroporator. Luciferase activity was measured 24 hours post‐transfection using the Dual‐Luciferase assay kit (Promega, Madison, WI, USA) according to the manufacturer's instructions. Experiments were repeated three times.

### 2.6 Lentivirus production and infection of cells

Full‐length pre‐miR‐149 and scramble‐miR‐149 were generated by PCR amplification from pUC18‐miR‐149 or pUC18‐scramble‐miR‐149 vector. The fragments were cloned into the lentiviral vector (LV5) under control of the EF‐1a promoter to construct the expression vectors LV5‐miR‐149 and LV5‐miR‐NC (Control). We co‐transfected the two lentiviral vectors with 3.5 μg PG‐P2‐REV/PG‐P3‐RRE and 1.5 μg PG‐P1‐VSVG plasmids into 293T packaging cells using Lipofectamine™ 2000. Lentivirus‐containing supernatants were harvested 48 hours after transfection and filtered through 0.22 μm cellulose acetate filters (Millipore, Billerica, MA, USA). Recombinant lentiviruses were concentrated by ultracentrifugation (2 hours at 50 000 ***g***).

For lentiviral infection, the culture medium was removed when the EC1 and EC9706 cells were at 60% confluence. The cells were washed gently with PBS and treated with the virus‐containing medium (MOI = 2) combined with Polybrene (5 μg/mL; Sigma, St Louis, MO, USA). Cells were grown in RPMI 1640 containing 10% FBS, with a change of medium every 48 hours. Then, cells were subdivided into three groups: transfected with NaCl (Blank), transfected with scrambled‐miR‐149 (miR‐NC) and transfected with pre‐miR‐149 (miR‐149).

### 2.7 CCK‐8 assay

Cells were transferred to 96‐well plate at a density of 1 × 10^4^ cells/well, with five replicate wells per group. Next, the cells were treated with 0‐30 μmol/L cisplatin (dissolved in NaCl, stored at −20°C). After 48 hours, the relative numbers of viable cells were detected using Cell Counting Kit‐8 reagents (CCK‐8; Dojindo, Japan). Results were recorded using a microplate reader (Elx800; BioTek, VT, USA), with the absorbance optical density at 450 nm, and the IC50 was calculated. The experiments were done in triplicate.

### 2.8 Flow cytometry assay

The cells were washed once with PBS, and then cultured in fresh medium containing 5 μg/mL 5‐FU or 0.5 μg/mL cisplatin. Cells were harvested at 48 hours post‐transfection by trypsinization, and resuspended at 10^6^ cells/mL in 1× binding buffer. After double staining with FITC‐Annexin V and propidium iodide (PI) using the FITC Annexin V Apoptosis Detection Kit I (BestBio, Shanghai, China), cells were analysed using a FACScan flow cytometer (BD Biosciences, USA) equipped with Cell Quest software (BD Biosciences).

### 2.9 Caspase3/7 activity assay

Cells from each treatment group were harvested at 48 hours post‐transfection and caspase activity was measured using a caspase activity assay kit (Beyotime, Haimen, China). Cellular extracts and substrates (Ac‐DEVD‐pNA) were kept in 96‐well plate for 2 hours at 37°C. Absorbance values were measured using a microplate reader at 405 nm (Infinite M200, Tecan, Switzerland).

### 2.10 Immunofluorescence microscopy

Immunofluorescence was performed to observe the expression level of γ‐H2AX in the nuclei of cells in different treatment groups. The cells were fixed with 4% paraformaldehyde for 15 minutes at room temperature and permeabilized with 0.1% Triton X‐100. After blocking in PBS containing 5% BSA for 1 hours, the cells were incubated with anti‐γ‐H2AX primary antibodies and stained with FITC‐conjugated secondary antibodies. Nuclei were stained with DAPI for 15 minutes. The mean number of immunostained nuclei per high‐power field was determined using immunofluorescence microscopy. Results are presented as the average of at least three fields.

### 2.11 DNA sequencing analysis

PCR‐amplified fragments were cloned into pGEM‐T vectors and transformed into Escherichia coli DH5α, which were grown at 37°C to mid‐log phase. The DH5α transformants were subjected to sequencing analysis at Sangon Biotech (Shanghai).

### 2.12 Surface plasmon resonance

The binding ability of miR‐149 and polβ was tested by Surface Plasmon Resonance (SPR) using a Biacore T200 instrument (Biacore, GE Healthcare). Biotinylated miR‐149 and miR‐NC were purchased from Sangon Biotech (Shanghai) and immobilized in channels 2 and 4, respectively, of BIAcore SA chips that were coated with streptavidin (GE Healthcare); channels 1 and 3 were left blank as references. Several fold dilutions of Wt‐polβ, C1134G‐polβ or Mt‐polβ mRNA fragments (Sangon Biotech Co., Ltd.) were injected at a flow rate of 20 mL/min. The binding time was 100 seconds, with dissociation times of 400 seconds. Binding and kinetics evaluations were analysed with BIAEVALUATION software (BIACORE). The binding data from the injection of different concentrations of analyte were globally fitted to a 1:1 binding model. Analyses with the same concentration series were done twice.

### 2.13 Statistical analysis

Statistical analysis was performed using SPSS 21.0 software. Data were expressed as the mean ± standard deviation. The *t*‐test was used for comparison of means from different samples. The follow‐up data were analysed using the Kaplan‐Meier method and log‐rank test. *P *<* *.05 was considered statistically significant.

## 3 RESULTS

### 3.1 polβ and miR‐149 are associated with chemotherapy sensitivity and survival time in EC patients

**Figure 1:**
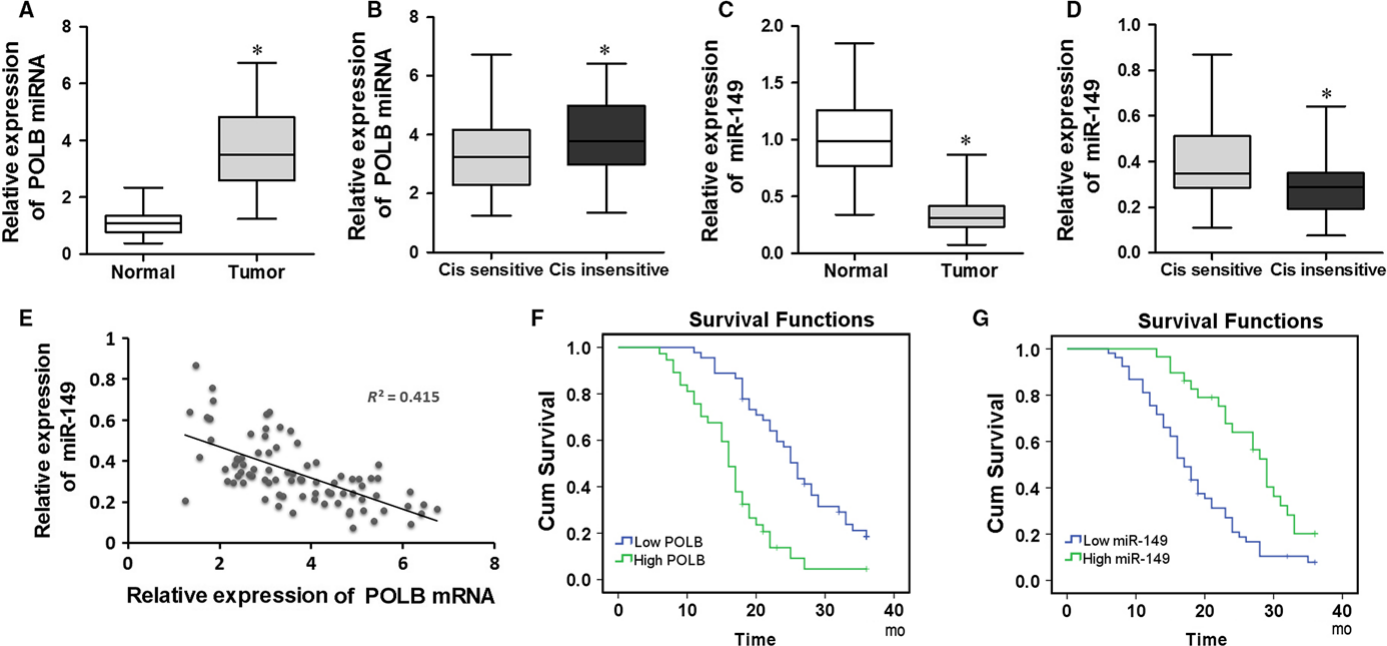
polβ and miR‐149 are associated with chemotherapy sensitivity and survival time in EC patients. A and B, The expression levels of polβ mRNA in EC specimens. HET‐1A cell line was used as untreated control. C and D, The expression levels of miR‐149 in EC specimens. HET‐1A cell line was used as untreated control. E, Correlation between polβ mRNA and miR‐149 in EC tumour tissues. F and G, Survival rates between the patients with high and low expression levels of polβ mRNA or miR‐149 in EC patients. *P < .05

qRT‐PCR assay was used to detect the expression levels of polβ mRNA and miR‐149 in EC specimens. Results showed that the expression levels of polβ mRNA in the tumour tissues was significantly higher than in adjacent non‐tumour tissues (*P *<* *.05, Figure 1A). In addition, the expression levels of polβ mRNA in the tumour tissues of cisplatin insensitive patients was higher than that of cisplatin sensitive patients (*P *<* *.05, Figure 1B). Conversely, the expression levels of miR‐149 in tumour tissues were significantly lower than in adjacent non‐tumour tissues (*P *<* *.05, Figure 1C). Furthermore, the expression levels of miR‐149 in the tumour tissues of cisplatin in‐sensitive patients were lower than that of cisplatin sensitive patients (*P *<* *.05, Figure 1D). We also investigated the correlation between polβ and miR‐149 expression, and found that polβ mRNA expression levels were increased in the EC tumour tissues, whereas miR‐149 expression was reduced, demonstrating a significant negative correlation (*R* ^2^ = 0.623, *P *<* *.05, Figure 1E).

The Kaplan‐Meier method was used to analyse the difference in survival rates between the patients with high and low expression levels of polβ mRNA or miR‐149 (median value was chosen as the boundary of high and low expression), based on follow‐up visits of EC patients. Patients with low expression levels of polβ mRNA or high miR‐149 survived longer than patients with high polβ mRNA or low miR‐149 (*P *<* *.05, Figure 1F,G).

### 3.2 Polβ is identified as a target gene of miR‐149 in EC1 and EC9706 cell lines

**Figure 2:**
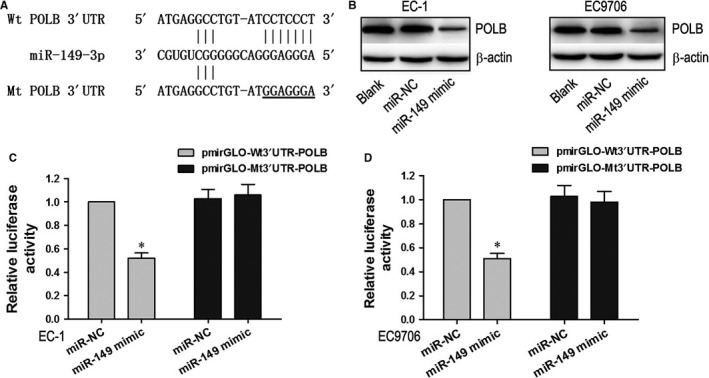
polβ is a target of miR‐149 in EC1 and EC9706 cell lines. A, The putative miR‐149 binding sequences for the polβ 3′UTR. B, polβ protein expression levels in miR‐149, Blank, and miR‐NC cells. C and D, Luciferase activity in EC1 and EC9706 cell lines following co‐transfection with miR‐149 and wild‐type 3′UTR polβ. *P < .05

Bioinformatics analysis by TargetScan and miRanda predicted that the 3′UTR of polβ contained binding sites for miR‐149 (Figure 2A). Figure 2B shows polβ expression in cells tranfected with miRNA alone (miR‐149 mimic or scrambled‐miR‐149 negative control). polβ protein expression levels in miR‐149 group cell was significant lower than that in Blank and miR‐NC groups (*P *<* *.05). To verify whether polβ is a direct target of miR‐149, we used a Dual‐Luciferase reporter system containing either the wild‐type or the mutant 3′UTR of polβ. There was no significant difference in luciferase activity between cells transfected with miR‐NC and those co‐transfected with miR‐149 mimic and mutant‐type 3′UTR of polβ (Figure 2C,D). However, the luciferase activity of cells co‐transfected with miR‐149 mimic and wild‐type 3′UTR of polβ was significantly decreased *(P *<* *.05, Figure 2C,D). These results indicate that miR‐149 negatively regulates polβ expression by directly binding to the putative binding sites in the 3′UTR.

### 3.3 Cisplatin‐induced proliferation inhibition was enhanced by miR‐149 in EC1 and EC9706 cell lines

**Figure 3:**
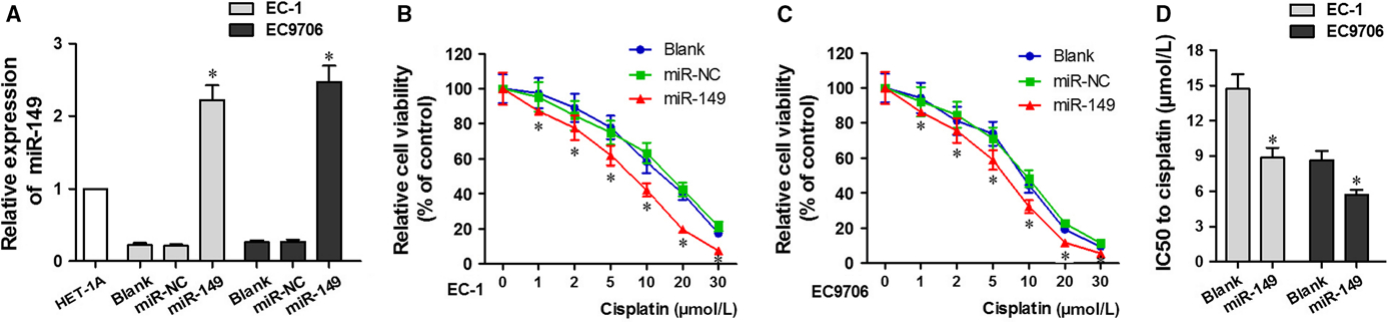
Cisplatin‐induced proliferation inhibition was enhanced by miR‐149 in EC1 and EC9706 Cell Lines. A, Relative miR‐149 expression in different cell lines. B and C, Cell viability in the miR‐149, Blank, and NC groups at different cisplatin doses in EC1 and EC9706 cell lines. D, IC50 of miR‐149 and Blank groups

The relative expression of miR‐149 in different cell lines is presented in Figure 3A. The expression of miR‐149 was higher in the miR‐149 group compared to the Blank and miR‐NC groups (*P *<* *.05). The cell survival rate curves are presented in Figure 3B,C. Cell viability in all groups decreased with increasing doses of cisplatin. The viability of cells in the miR‐149 group was significantly lower than that of the Blank and NC groups, across cisplatin doses (*P *<* *.05, Figure 3B,C). The IC50 of cisplatin in EC1 and EC9706 cells was calculated, and this is shown in Figure 3D. The IC50 of cisplatin in the miR‐149 cells was lower than that of the Blank and NC groups (*P *<* *.05).

### 3.4 Cisplatin‐induced apoptosis was enhanced by miR‐149 in EC1 and EC9706 cell lines

**Figure 4:**
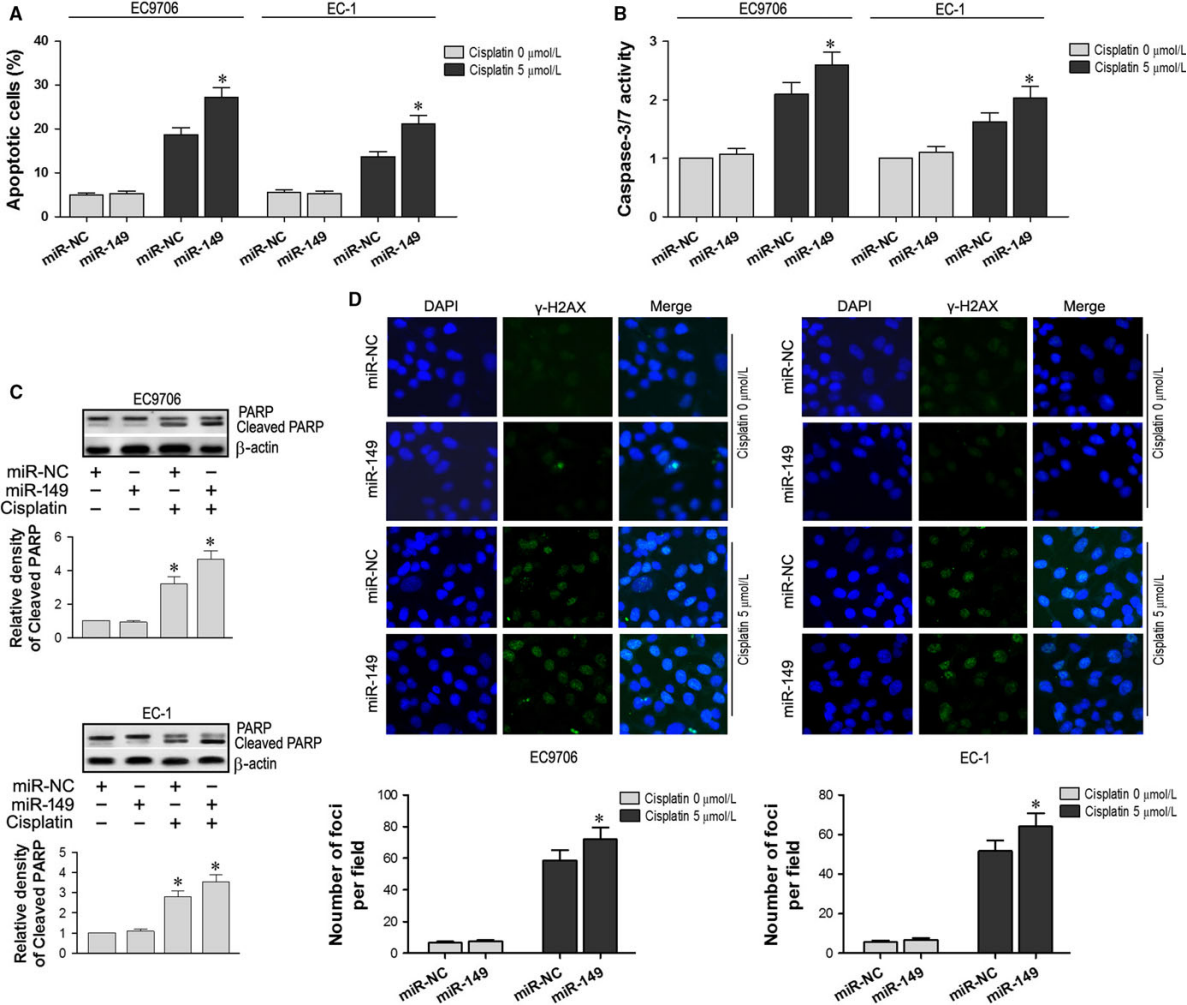
Cisplatin‐induced apoptosis was enhanced by miR‐149 in EC1 and EC9706 cell lines. A, Apoptosis in miR‐149 and miR‐NC cells following treatment with cisplatin (*P *<* *.05). B, Caspase 3/7 activity in miR‐149 and miR‐NC cells (*P *<* *.05). C, Cleaved PARP expression in miR‐149 cells treated with cisplatin (*P *<* *.05). D, The expression level of γ‐H2AX in the nuclei of miR‐149 and miR‐NC cells cultured with and without cisplatin. *P <* *.05

Flow cytometry results indicated that treatment with cisplatin increased apoptosis in both the miR‐NC and miR‐149 cells. In addition, apoptosis in the miR‐149 cells was significantly greater than that in the miR‐NC cells (*P *<* *.05; Figure 4A). In addition, after treatment with cisplatin, caspase 3/7 activity in the miR‐149 cells was significantly higher than in the miR‐NC cells (*P *<* *.05; Figure 4B). Western blotting showed that the expression of cleaved PARP was also significantly increased in miR‐149 cells treated with cisplatin (*P *<* *.05; Figure 4C). Immunofluorescence microscopy results indicated that the expression level of γ‐H2AX in the nuclei of miR‐149 cells treated with cisplatin was significantly higher than that of miR‐NC cells treated with cisplatin, and miR‐NC or miR‐149 cells without cisplatin (*P *<* *.05; Figure 4D). These results indicated that miR‐149 could enhance the apoptotic effects of cisplatin in EC1 and EC9706 cell lines.

### 3.5 Expression of polβ restored the function of miR‐149

**Figure 5:**
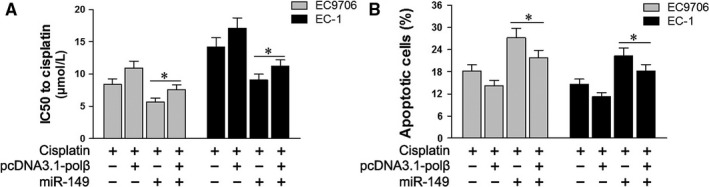
Expression of polβ restored the function of miR‐149. A, IC50 values of cisplatin on the proliferation of EC1 and EC9706 cell lines following co‐transfection of pcDNA3.1‐polβ and miR‐149. B, Apoptosis in EC1 and EC9706 cell lines treated with cisplatin following co‐transfection of pcDNA3.1‐polβ and miR‐149. *P <* *.05

As discussed above, the exogenous expression of miR‐149 enhanced the anti‐proliferative and pro‐apoptotic effects of cisplatin in EC1 and EC9706 cell lines (Figures 3 and 4). Therefore, we exogenously expressed recombinant polβ lacking the 3′ UTR sequence (pcDNA3.1‐polβ) in EC1 and EC9706 cells. The IC50 values of cisplatin indicated that co‐transfection of pcDNA3.1‐polβ and miR‐149 abrogated the effects of miR‐149 on the proliferation of EC1 and EC9706 cell lines treated with cisplatin (*P *<* *.05, Figure 5A). Flow cytometry results showed that co‐transfection of pcDNA3.1‐polβ and miR‐149 abrogated the apoptotic effects of miR‐149 on EC1 and EC9706 cell lines that treated with cisplatin (*P *<* *.05, Figure 5B).

### 3.6 miR‐149 did not regulate the C1134G variant of polβ in EC tissues

**Figure 6:**
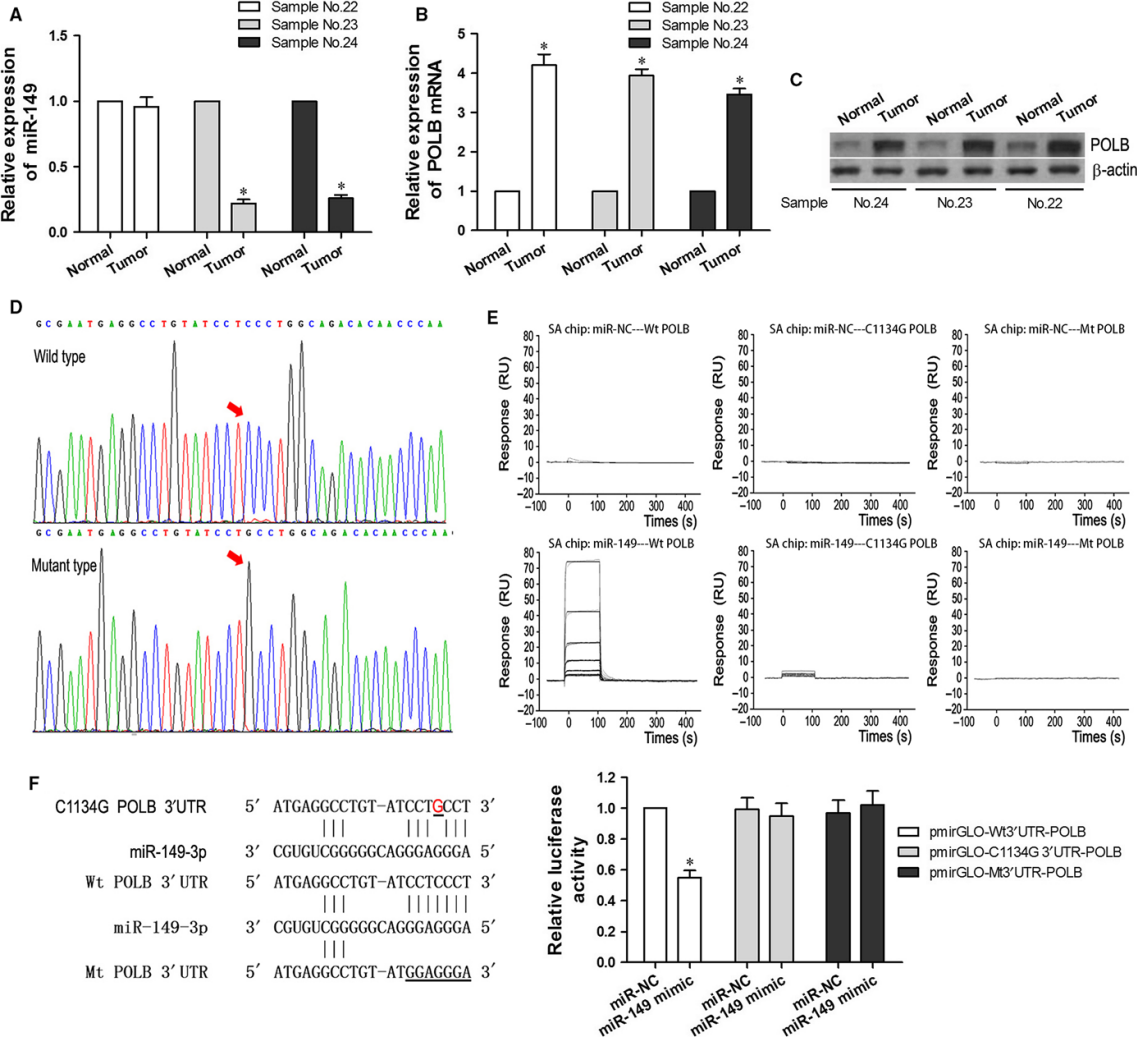
miR‐149 did not Regulate C1134G Variant of polβ in EC Tissues. A, miR‐149 expression levels in the tumour tissue of sample No. 22 and adjacent non‐tumour tissue. B and C, polβ mRNA and protein levels in the tumour tissue of sample No. 22 and adjacent non‐tumour tissue. (**D**) polβ DNA sequencing result of sample No. 22. E, Affinity curves demonstrating the binding affinities of miR‐149 and polβ variants. F, Luciferase activity in EC9706 cell lines following co‐transfection with miR‐149 and C1134G 3′UTR of polβ. *P < .05

We have shown that the expression level of miR‐149 in the tumour tissues was significantly lower than in adjacent non‐tumour tissues, and that polβ mRNA expression in tumour tissues was significantly higher than in adjacent non‐tumour tissues (*P *<* *.05, Figure 1A,C). Interestingly, in one sample (No. 22), the expression levels of miR‐149 in the tumour tissue were not lower, and polβ expression in the tumour tissue was higher, compared to the adjacent non‐tumour tissue (Figure 6A‐C). Using a DNA sequencing assay, we analysed the polβ DNA sequence of sample No. 22 and identified a novel homozygous C to G point mutation at nucleotide 1134 (Figure 6D).

To further analyse the binding properties of polβ with miR‐149, surface plasmon resonance analysis was performed. BIAcore analysis confirmed that the binding affinity of miR‐149 and wild‐type polβ is stronger than that of the miR‐149 and C1134G variants of polβ, and much stronger than that of the miR‐149 and mutant‐type polβ, or the miR‐NC and polβ variants (Figure 6E).

To verify the relationship between the C1134G variant of polβ and miR‐149, we conducted a Dual‐Luciferase reporter assay in polβ^−/−^ EC9706 cells. The luciferase activity of cells co‐transfected with miR‐149 mimic and wild‐type 3′UTR of polβ was significantly decreased (*P *<* *.05, Figure 6F). Conversely, there was no significant change in the luciferase activity of cells co‐transfected with miR‐149 and mutant‐type polβ, or the miR‐NC and polβ variants. These results indicate that miR‐149 may be unable to regulate the C1134G variant of polβ in EC tissues and EC9706 cells.

## 4 DISCUSSION

EC is a major cause of cancer‐related deaths worldwide. Although methods for the diagnosis and treatment of EC have advanced, the disease continues to have a poor prognosis due to invasion and early stage metastasis.^30^, ^31^ Due to the difficulty of surgical intervention and its potential complications, most patients choose to undergo palliative treatments such as chemotherapy. However, the clinical efficacy of chemotherapy is inadequate; the 5‐year survival rate is only 10%‐30%, and the rates of local uncontrolled tumour growth and recurrence range from 60%‐80%.^32^, ^33^ Therefore, researchers are currently focusing on increasing the efficacy of chemotherapy in EC. In recent years, studies have identified a variety of genes whose expression products may affect tumour response to chemotherapy; examples include cell cycle regulatory genes, apoptotic genes, and DNA damage repair proteins.

DNA polymerase β (polβ) is a key enzyme in the DNA damage repair system. This system serves as a crucial factor in maintaining genome integrity and stability, in addition to modulating the chemotherapy sensitivity of tumour cells. Many studies demonstrated that the polβ is required for cisplatin sensitivity.^34^, ^35^ Not surprisingly, polβ has become a research hot spot worldwide.

miRNAs have been estimated to regulate up to 30% of human genes and control a variety of cellular processes.^36^ Recent studies have shown that miRNAs are dysregulated in various cancers, and that their expression is relevant to a diverse array of tumours.^37^, ^38^

In this study, we determined that the expression levels of polβ mRNA in tumour tissues were significantly higher than that in adjacent non‐tumour tissues. Interestingly, the expression levels of polβ mRNA in the tumour tissues of cisplatin insensitive patients were higher than in cisplatin sensitive patients, and the expression level of miR‐149 in the EC tumour tissues was inverse to that of polβ. Our study revealed that polβ mRNA expression levels were increased, where miR‐149 mRNA expression levels were reduced, demonstrating a negative correlation. Kaplan‐Meier method results showed that patients with low expression levels of polβ mRNA or high miR‐149 survived longer than patients with high polβ mRNA or low miR‐149. Bioinformatics analysis and dual‐luciferase reporter assay predicted that the 3′UTR of polβ contained binding sites for miR‐149, and that miR‐149 negatively regulates polβ expression by directly binding to the 3′UTR. CCK‐8 assay indicated that miR‐149 could enhance the anti‐proliferative effects of cisplatin in EC1 and EC9706 cell lines. Flow cytometry, caspase 3/7 activity, PARP cleavage, and immunofluorescence microscopy results of γ‐H2AX in the nucleus indicated that miR‐149 could enhance the pro‐apoptotic effects of cisplatin in EC1 and EC9706 cell lines. In addition, we also showed that the expression of polβ lacking the 3′UTR sequence could override the anti‐proliferative and pro‐apoptotic functions of miR‐149, suggesting that miR‐149 negatively regulates polβ expression by binding to its 3′UTR. Surface plasmon resonance results also showed that the miR‐149 could bind with wild‐type polβ.

Interestingly, the expression levels of miR‐149 and polβ in sample No. 22 were abnormal. The miR‐149 expression level in the tumour tissue was not lower, and polβ expression in the tumour tissues was higher, compared to the adjacent non‐tumour tissue. DNA sequencing assay was used to analyse the polβ DNA sequence of sample No. 22, and revealed a novel homozygous C to G point mutation at nucleotide 1134 in polβ DNA. Dual‐luciferase reporter assay results indicated that miR‐149 may be unable to regulate the C1134G variant of polβ in EC tissue and EC9706 cells.

In conclusion, this study confirms that miR‐149 may enhance cisplatin sensitivity in EC cell lines by targeting polβ. In addition, we identified a new variant of polβ (C1134G) and found that miR‐149 may be unable to regulate the C1134G variant of polβ. Based on these findings, potential drugs could be developed with a focus on enhanced sensitivity of EC patients to chemotherapy.

## CONFLICTS OF INTEREST

The authors declare no conflict of interest.
